# Language and culture modulate online semantic processing

**DOI:** 10.1093/scan/nsv028

**Published:** 2015-03-12

**Authors:** Ceri Ellis, Jan R. Kuipers, Guillaume Thierry, Victoria Lovett, Oliver Turnbull, Manon W. Jones

**Affiliations:** ^1^Bangor University, LL58 2AS Bangor, UK,; ^2^University of Stirling, FK9 4LA Stirling, UK, and; ^3^Swansea University, SA2 8PP Swansea, UK

**Keywords:** linguistic relativity, bilingualism, culture, semantics

## Abstract

Language has been shown to influence non-linguistic cognitive operations such as colour perception, object categorization and motion event perception. Here, we show that language also modulates higher level processing, such as semantic knowledge. Using event-related brain potentials, we show that highly fluent Welsh–English bilinguals require significantly less processing effort when reading sentences in Welsh which contain factually correct information about Wales, than when reading sentences containing the same information presented in English. Crucially, culturally irrelevant information was processed similarly in both Welsh and English. Our findings show that even in highly proficient bilinguals, language interacts with factors associated with personal identity, such as culture, to modulate online semantic processing.

## INTRODUCTION

Recent research has shown that language affects basic cognitive functions such as perception and object categorization (Thierry *et al.*, 2009; Boutonnet *et al.*, 2012), thus making large strides in resolving the contentious debate surrounding the influence of language on human cognition (Whorf, 1956; Lakoff, 1987; Hunt and Agnoli, 1991; Bowerman and Levinson, 2001; Levinson, 2003). At higher levels of conceptual representation, it is commonly accepted that the semantic level is shared across all languages spoken by an individual (De Groot, 1992; Kroll and Stewart, 1994; La Heij *et al.*, 1996; Van Hell and De Groot, 1998; Gollan and Kroll, 2001). However, recent evidence suggests that the language of operation also affects higher level representations, as is the case in the domain of lexically driven semantic associations (Boutonnet *et al.*, 2014) and motion conceptualization (Kersten *et al.*, 2010; Athanasopoulos *et al.*, 2015). Here, we provide the first empirical, neurophysiological evidence that the language in which someone operates interacts with personal factors such as cultural identity to modulate online semantic processing during sentence comprehension.

Behavioural studies have shown that language shapes conceptual information. Abstract linguistic idiosyncrasies, such as arbitrary male–female gender marking, influence the perception of semantically gender–neutral objects (Boroditsky, 2001; Boroditsky *et al.*, 2003), and the effect of factors relating to personhood, such as cultural biases induced by native personal pronouns, is heightened when information is presented in the native language (Danziger and Ward, 2010; Ogunnaike *et al.*, 2010). However, such findings remain sparse and limited to single nouns and pronouns. The link between language and personhood, which is a defining feature of culture, may therefore be redolent of phenomena such as the implicit activation of racial attitudes and biases [see Fiedler *et al.*, 2006, for a critique of the Implicit Association Task (IAT)], but it remains unknown whether the languages spoken by an individual each interact differently with culture to affect ‘comprehension’. This distinction is important, in that evocation of attitudes is generally conceived as an automatic, ‘knee-jerk’ reaction to a stimulus, whereas comprehension refers to semantic analysis, synthesis and understanding of linguistic information.

In this study, we tested whether language and cultural factors may interact to modulate sentence comprehension in fluent, early adult Welsh–English bilinguals. We recorded electrophysiological responses in bilingual participants reading Welsh and English sentences. Half of the sentences in each language contained culturally relevant information; the other half referred to culturally non-relevant facts, that is, generic semantic knowledge. Furthermore, and in order to implement a suitable cognitive task, half of the sentences formed a true premise and the other half a false one (Table 1). Semantic processing was indexed by the amplitude of the N400 wave of the event-related potential (ERP) elicited by the sentence-final word, identical between experimental conditions. N400 amplitude is modulated by the extent to which the target word fits the semantic context in which it is presented, with increasing negative amplitude indexing greater energy required for semantic integration (Kutas and Federmeier, 2011). Current theorizing on N400 modulation implicates lexical retrieval from long-term memory, which is facilitated by top-down context information from the preceding sentence fragment (Van Berkum, 2009; Brouwer *et al.*, 2012). In the current experiment, participants pressed buttons to indicate whether each presented statement was true or false, thus providing a direct measure of sentence comprehension. We predicted reduced N400 amplitudes for words completing a true statement as compared with these same words completing a false statement by virtue of the fact that true statements are naturally more expected than false ones. We further hypothesized a differential effect of language for culturally relevant content, and thus expected to find an interaction between language and cultural relevance. More specifically, we anticipated a greater true–false N400 disparity for information about Wales or Welsh people presented in Welsh as compared with the same information presented in English. Such an interaction would indicate that semantic processing is indeed different in the two languages insofar as they shed a different light on culturally relevant information.

**Table 1:** Experimental design and example of a sentence set

Sentence	Premise	Cultural relevance
a—Presented in English^a^		
Every single Welsh child can sing in ***tune***	False	Relevant
Opera at the National Welsh Theatre is always in ***tune***	True	Relevant
Good quality antique instruments always stay in ***tune***	False	Non-relevant
Before a professional concert, a piano is always in ***tune***	True	Non-relevant
b—Presented in Welsh^a^		
The National Welsh Theatre is the only venue where opera is in ***tune***	False	Relevant
A lot of Welsh children can sing in ***tune***	True	Relevant
The piano is the only instrument that stays in ***tune***	False	Non-relevant
Old instruments are quite likely to be out of ***tune***	True	Non-relevant

^a^Counterbalanced across participants.

## MATERIALS AND METHODS

### Participants

Eighteen balanced Welsh–English bilinguals with normal or corrected vision (1 male, 17 women; *M* = 22.06 years, s.d. = 5.03) were included in the analysis. Five participants were excluded because they had too few artefact-free epochs per condition. Participants self-reported that they were L1 Welsh speakers, having been exposed to English from an early age (*M* = 4.22 years, s.d. = 2.88). The sample reported, on average, 66% L1 and 34% L2 usage in everyday interactions, including bilingual educational instruction. Ethical approval was granted by the School of Psychology, Bangor University ethics committee, and participants gave written consent.

### Stimuli and procedure

A total of 40 English sentence sets and 40 Welsh translation equivalents were constructed. In each language, each set consisted of 8 sentences ending in the same final word. Participants were presented with 4 sentences from the English set, and 4 different sentences from the Welsh set (Table 1). Thus, for any given participant, each experimental sentence was not repeated, not even by way of a translation equivalent. Of these sentences, the language factor (English *vs* Welsh) was crossed with a cultural relevance factor (relevant *vs* non-relevant) and a truth-value factor (true *vs* false). The procedure included two important counterbalancing features: (i) the truth value (true *vs* false) of sentences containing a particular referent (e.g. ‘instruments’) was inverted between languages of presentation and (ii) the language of sets a and b was fully rotated between participants.

In a separate pre-test, 20 participants who did not take part in the experiment proper were asked to complete the sentences with the first 3 words that came to mind. If one of the completions matched our experimental sentences, a score of 1 was given. All other answers were given a score of 0. When scores were averaged across sentences, a cloze probability of 42% was obtained, which was above our threshold of 40% (Coulson *et al.*, 2006), and there was no significant difference between conditions (*P* > 0.05). Sentence-final target words were controlled for written frequency, word and syllable length [‘Cronfa Electroneg o Gymraeg’ (Welsh), Ellis *et al.*, 2001; CELEX lexical database (English), Baayen *et al.*, 1993]. Each participant thus read 320 sentences in total presented in 8 experimental blocks.

Stimuli were presented in white courier new 18 point font on a black background on a 19-inch cathod ray tube (CRT) monitor with a refresh rate of 75 Hz. The first clause of each sentence was presented all at once and reading was self-paced, followed by single word presentations in the centre of the screen for 200 ms with an inter-stimulus interval of 500 ms (so as to prevent eye movements upon presentation of the final word). Presentation order was pseudorandomized such that participants would not encounter the same final word within the same block. Following each sentence, participants made a yes/no judgement regarding the truth value of each statement.

### ERP recording

Electrophysiological data were recorded from 64 Ag/AgCl electrodes according to the extended 10–20 convention, referenced to the Cz electrode at a rate of 1 kHz. Impedances were kept below 5 kΩ. Electroencephalogram activity was filtered online with a band-pass filter between 0.1 and 200 Hz and offline using a low-pass, zero phase shift digital filter with a cut-off frequency of 20 Hz. Eye blink artefacts were corrected mathematically using the procedure proposed by Gratton *et al.* (1983), and remaining artefacts were removed manually upon visual inspection of the data. Epochs ranged from −100 to 1000 ms after final word onset. Epochs with activity exceeding ± 75 µV at any electrode site over the scalp were discarded. Baseline correction was performed in reference to pre-stimulus activity and individual averages were digitally re-referenced to the global average reference.

## RESULTS

Analyses were conducted on 79% of the data, that is, sentences that were accurately verified as true or false (cf. Martin *et al.*, 2014). Repeated measures ANOVAs were conducted with language (Welsh *vs* English), cultural relevance (relevant *vs* non-relevant) and Truth value (true *vs* false) as independent variables.

### Behavioural data

**Fig. 1:**
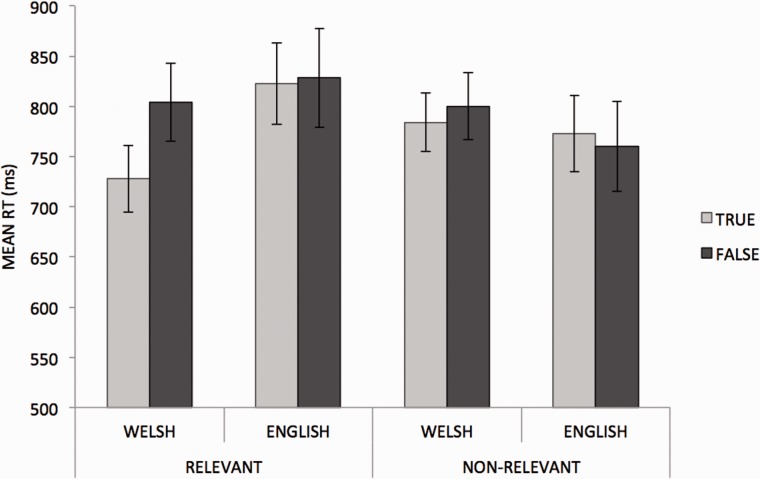
Mean RTs (ms) for correct true/false responses to culturally relevant or non-relevant statements presented in Welsh or English. Error bars represent SEM.

Analysis of variances (ANOVAs) on reaction time data yielded no main effects of cultural relevance (*F*_(1, 17)_ = 1.15, *P* > 0.05), language (*F*_(1, 17)_ = 0.35, *P* > 0.05) or truth value (*F*_(1, 17)_ = 1.23, *P* > 0.05). A language by truth value interaction (*F*_(1, 17)_ = 4.81, *P* = 0.042) showed that in the case of Welsh sentences, true statements were responded to more quickly than false ones, whereas statements in English were responded to with similar speed independent of truth value. There was also a language by cultural relevance interaction (*F*_(1, 17)_ = 10.71, *P* = 0.004) such that culturally relevant statements were responded to more quickly than non-relevant statements when sentences were presented in Welsh, but no such difference was found for statements in English. No other interactions emerged from the reaction time data (Figure 1). A correlation analysis by subjects revealed no evidence of a speed–accuracy trade-off (*r*(1, 18) = 0.09, *P* = 0.73).

### Electrophysiological data

**Fig. 2:**
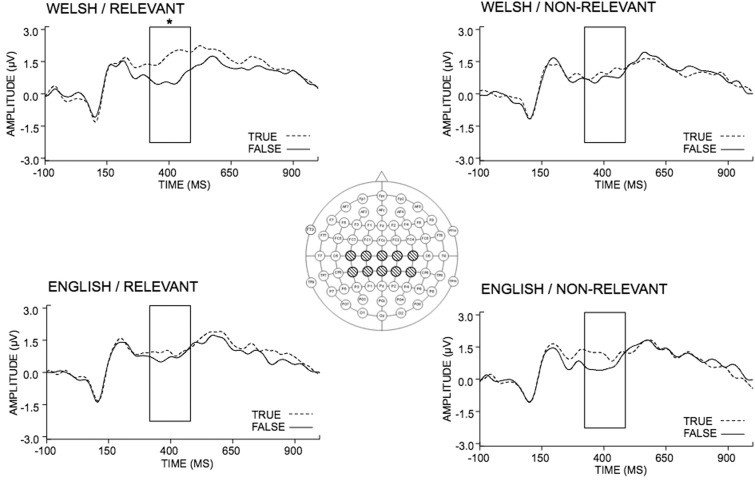
ERPs (µV) elicited by true/false sentences containing culturally relevant or culturally non-relevant information and presented in either Welsh or English. The asterisk indicates the window of analysis in which mean ERP amplitudes significantly differed between conditions (340–450 ms post-stimulus).

We analysed ERP amplitudes over 10 electrodes over which the N400 is known to be maximal (linear derivation of Cz, C1, C2, C3, C4, CPz, CP1, CP2, CP3, CP4; Kutas and Hillyard, 1980a,b, 1984; Martin *et al.*, 2009; Hoshino and Thierry, 2012; see also Kutas and Federmeier, 2011; Luck, 2014, p. 52) (Figure 2). As expected, there was a main effect of truth value (*F*_(1, 17)_ = 19.65, *P* < 0.001), such that the N400 was reduced in amplitude for true relative to false statements and no other main effects (cultural relevance: *F*_(1, 17)_ = 1.71, *P* > 0.05; language: *F*_(1, 17)_ = 1.43, *P* > 0.05) or two-way interactions (language and truth: *F*_(1, 17)_ = 1.35, *P* > 0.05; language and culture: *F*_(1, 17)_ = 2.28, *P* > 0.05; truth and culture: *F*_(1, 17)_ = 1.34, *P* > 0.05) emerged.

**Fig. 3:**
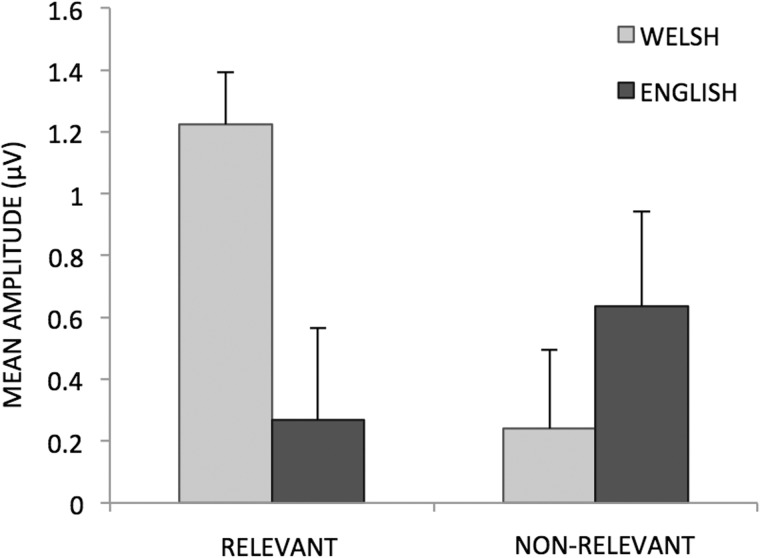
Mean amplitude (µV) of the N400 effect (truth–false) for culturally relevant and non-relevant statements presented in Welsh or English. Error bars represent SEM.

Critically, we found a significant three-way interaction among language, cultural relevance and truth value (*F*_(1, 17)_ = 6.01, *P* = 0.025). Planned comparisons on the N400 effect (true–false) in the different conditions showed that the N400 was significantly larger for Welsh than English in the culturally relevant conditions (*t*(17) = 3.12, *P* = 0.006; Figures 2 and 3), whereas no language difference was found for culturally non-relevant sentences (*t*(17) = −0.95, *P* > 0.05).

Overall, our results show that for statements containing information about Wales, the N400 effect is larger when these statements are presented in Welsh than in English. Importantly, this finding is not due to a generic/overall preference for the Welsh language because no language differences were found for general statements.

## Discussion

We investigated whether the language we speak can influence the way in which we understand detailed, sentence-level information, using the N400 ERP wave as an index. Our findings indicate that language interacts with cultural identity during semantic processing. More specifically, we show that true, culturally relevant information about Wales is integrated with more ease when it is presented in Welsh than in English, even though English translation equivalents are supposed to convey identical information. In contrast, culturally non-relevant information is not processed differently between languages. To our knowledge, this is the first demonstration that information intimately linked with the native language (e.g. because of the emotional context in which knowledge is acquired) is processed more readily in that language than in another language acquired subsequently. Language therefore affects cognition even at the subtlest levels of semantic knowledge.

It is noteworthy that the ERP modulations reflecting online semantic processing at 400 ms post stimulus were broadly consistent with behavioural reaction time (RT) sentence-verification data acquired simultaneously: Factually correct and culturally relevant information yielded shorter RTs, supporting the interpretation of easier semantic processing when language and cultural references are aligned.

We believe that these findings significantly extend insights into linguistic relativity effects. Previous electrophysiological studies have shown an influence of language on basic cognitive functions such as perception and object categorization (Thierry *et al.*, 2009; Athanasopoulos *et al.*, 2010; Boutonnet *et al.*, 2013). Furthermore, emotional words have been shown to have a different resonance or impact on the first and second language of bilinguals (Dewaele, 2004; Wu and Thierry, 2012), but the effect of language on higher order levels of semantic processing such as cultural relevance has seldom been observed. Recent studies using the IAT suggest that language and culture interact in complex ways (Danziger and Ward, 2010; Ogunnaike *et al.*, 2010). In both studies, bilinguals showed faster responses to native personal pronouns paired with positive adjectives (such as ‘good’) when the task was performed in the native relative to the non-native language. The authors concluded that language serves as a cue for the activation of certain racial biases (Briley *et al.*, 2005; Danziger and Ward, 2010).

Our study also shows that the languages spoken by a bilingual do not equally convey cultural mores, such that a statement may not be understood in the same way at all depending on its reference to the speaker’s native culture. Thus, linguistic relativity is not confined to automatic reactions defining attitudes, prejudice or belief (Briley *et al.*, 2005). ‘False’ statements in our study involved fairly subtle misinformation, conforming to folk wisdom and national pride (e.g. ‘Welsh collies are the most intelligent breed of dog’). However, the N400 for false, culturally relevant statements presented in Welsh was not attenuated in the same way as true, culturally relevant statements presented in Welsh. Thus, our findings suggest that language specifically influences online processing of real, verifiable semantic knowledge when it relates to culturally relevant information. Importantly, any modulation of the N400 in this study must have originated from the language used, because the semantic sentence context and critical final word were identical across language conditions (Kutas and Federmeier, 2011). We also note here that the duration of the N400 was sustained beyond the usual range (typically ∼250–550 ms, e.g. Kutas and Hillyard, 1980b; Hagoort *et al.*, 2004), which is perhaps to be expected given the relative subtlety of our true/false manipulation, leading to prolonged content evaluation or decision-making processes (Osterhout and Nicol, 1999; Hagoort, 2003; Hagoort *et al.*, 2003; Martín-Loeches *et al.*, 2006).

To conclude, our study provides the first neurophysiological demonstration that the language we speak interacts with personal factors such as cultural identity to modulate online semantic processing during sentence comprehension, one of the most sophisticated cognitive abilities of the human brain. The mechanism underlying these effects remains unknown, but is likely to involve episodic memory and the limbic system, both of which are known to be shaped by one’s cultural experience (Marian and Neisser, 2000; Schrauf, 2000; Marian and Kaushanskaya, 2004; Danziger and Ward, 2010). Future studies will hopefully shed more light on the existence and permanence of these effects across development and in bilingual adults with varying degrees of proficiency.
